# Peripheral Electrical Stimulation Modulates Cortical Beta-Band Activity

**DOI:** 10.3389/fnins.2021.632234

**Published:** 2021-03-25

**Authors:** Laura J. Arendsen, Robert Guggenberger, Manuela Zimmer, Tobias Weigl, Alireza Gharabaghi

**Affiliations:** ^1^Institute for Neuromodulation and Neurotechnology, University of Tübingen, Tübingen, Germany; ^2^Department of Anesthesiology and Intensive Care Medicine, University Hospital Bonn, Bonn, Germany

**Keywords:** sensorimotor rhythm, state-dependent stimulation, nociception, peripheral electrical stimulation, electroencephalography

## Abstract

Low-frequency peripheral electrical stimulation using a matrix electrode (PEMS) modulates spinal nociceptive pathways. However, the effects of this intervention on cortical oscillatory activity have not been assessed yet. The aim of this study was to investigate the effects of low-frequency PEMS (4 Hz) on cortical oscillatory activity in different brain states in healthy pain-free participants. In experiment 1, PEMS was compared to sham stimulation. In experiment 2, motor imagery (MI) was used to modulate the sensorimotor brain state. PEMS was applied either during MI-induced oscillatory desynchronization (concurrent PEMS) or after MI (delayed PEMS) in a cross-over design. For both experiments, PEMS was applied on the left forearm and resting-state electroencephalography (EEG) was recording before and after each stimulation condition. Experiment 1 showed a significant decrease of global resting-state beta power after PEMS compared to sham (*p* = 0.016), with a median change from baseline of −16% for PEMS and −0.54% for sham. A cluster-based permutation test showed a significant difference in resting-state beta power comparing pre- and post-PEMS (*p* = 0.018) that was most pronounced over bilateral central and left frontal sensors. Experiment 2 did not identify a significant difference in the change from baseline of global EEG power for concurrent PEMS compared to delayed PEMS. Two cluster-based permutation tests suggested that frontal beta power may be increased following both concurrent and delayed PEMS. This study provides novel evidence for supraspinal effects of low-frequency PEMS and an initial indication that the presence of a cognitive task such as MI may influence the effects of PEMS on beta activity. Chronic pain has been associated with changes in beta activity, in particular an increase of beta power in frontal regions. Thus, brain state-dependent PEMS may offer a novel approach to the treatment of chronic pain. However, further studies are warranted to investigate optimal stimulation conditions to achieve a reduction of pain.

## Introduction

In various pain conditions evidence has been found for central sensitization (Coderre et al., 1993; Banic et al., 2004; Latremoliere and Woolf, 2009; Woolf, 2011). Central sensitization broadly refers to hypersensitivity of the central nociceptive system, i.e., greater spinal excitability. Moreover, supra-spinal regions (the brainstem and higher brain centers) are important modulators of spinal excitability (Gwilym et al., 2009; Ossipov et al., 2010; Heinricher, 2016). Thus, modulating the excitability of the central nociceptive system offers a promising target for neurostimulation techniques to reduce pain.

Peripheral electrical stimulation (PES) is commonly used to facilitate motor rehabilitation and treat pain via the induction of plastic changes in corticospinal excitability (Klein et al., 2004; Chipchase et al., 2011; Dimyan and Cohen, 2011). For example, low frequency PES has been demonstrated to reduce pain perception and the spinal and cortical response to nociceptive stimuli (Jung et al., 2009, 2012; Rottmann et al., 2010). However, evidence for the clinical efficacy of PES interventions such as transcutaneous electrical nerve stimulation (TENS), a commonly used PES intervention to treat pain, is inconclusive (Catley et al., 2015; Johnson et al., 2015), leaving room for improvement.

Brain state-dependent stimulation, a novel development in the field of neurostimulation, could be of interest for the application of PES to reduce pain. Motor imagery (MI), the cognitive task of imagining a movement, is commonly used to modulate the sensorimotor brain state. MI results in sensorimotor desynchronization of alpha and beta oscillations (Fadiga et al., 1999; Pfurtscheller et al., 2006) and has a top-down influence on cortico-spinal excitability (Oishi et al., 1994; Li et al., 2004; Aoyama and Kaneko, 2011). Using MI to modulate the sensorimotor brain state, state-dependency of transcranial magnetic stimulation (TMS) and PES has been demonstrated for the motor system (Kraus et al., 2016a,b, 2018; Guggenberger et al., 2018, 2020; Ziegler et al., 2019). Stimulation applied concurrently with MI enhanced the modulation of corticospinal excitability, as reflected by an increase of the amplitude of the motor-evoked potential (MEP) (Saito et al., 2013; Kaneko et al., 2014; Kraus et al., 2016a,b). This was not the case when stimulation was applied after MI. Moreover, only when combined TMS and peripheral stimulation was applied concurrently with MI, a significant modulation of corticospinal excitability was present; this was not the case when the combined stimulation was applied after MI (Kraus et al., 2018). Thus, applying PES sensorimotor state-dependently may offer an opportunity to optimize the efficacy of low-frequency PES to modulate the excitability of the central nociceptive system and reduce pain. However, whereas previous work has predominantly examined the impact of MI combined with PES on cortico-spinal excitability of the motor system, the influence of MI and PES on the cortical response, especially in the context of nociceptive processing, is less understood.

The type of electrode used for PES is also of importance; the type of electrode influences the effect of PES on the nociceptive system (Steenbergen et al., 2012; Mücke et al., 2014). Larger surface electrodes conventionally used for TENS affect deeper tissues and activate tactile afferents. One of the main theories used to explain the effects of TENS on pain is the gate control theory of pain (Melzack and Wall, 1965). This theory proposes that a type of “gate” exists in the dorsal horn of the spinal cord that controls the transmission of large (non-nociceptive) and small (nociceptive) sensory afferents to the brain. This gate can be opened by noxious stimuli and closed by non-noxious stimuli. Following this, stimulation of large diameter tactile afferents by TENS it thought to reduce pain by inhibiting the transmission of noxious information in the spinal cord (Sluka and Walsh, 2003; Johnson et al., 2015).

In contrast, concentric electrodes preferentially activate nociceptive afferents in superficial skin layers (Kaube et al., 2000; Mücke et al., 2014). The use of repetitive electrical stimulation targeting the nociceptive fibers is thought to inhibit nociceptive processing and reduce pain via a long-term depression (LTD)-like phenomenon. Repetitive activation of synaptic connections can lead to long-term potentiation (LTP) or LTD of synaptic transmission (Madison et al., 1991; Siegelbaum and Kandel, 1991). This also applies to the transmission of noxious input. For example, it has been demonstrated that repetitive stimulation of nociceptive Aδ fibers produces LTD of C-fiber-evoked field potentials in rats (Liu et al., 1998). In humans, the induction of an LTD-like modulation of nociceptive processing and pain perception has also been demonstrated with a conditioning protocol consisting of low-frequency electrical stimuli. Using a concentric electrode, low-frequency PES has been shown to lead to a prolonged reduction of experimentally induced pain (Klein et al., 2004) that is accompanied by a reduction of the cortical evoked response to pain (Jung et al., 2009, 2012).

More recently, a new type of PES electrode has been developed that contains a matrix or grid of pin electrodes (Bomedus GmbH, Germany). Like the concentric electrode, the matrix electrode is designed to preferentially activate nociceptive afferents in the superficial skin layers, but with its larger size it allows for the stimulation of a larger skin area (Mücke et al., 2014). Compared to the concentric electrode, the matrix electrode was particularly effective in reducing deep pain sensitivity (Mücke et al., 2014). However, the cortical effects of low-frequency PES with the matrix electrode (PEMS) have not been investigated yet.

Oscillatory neural activity has been identified as a promising target for the development of novel pain therapies (Jensen et al., 2008). Chronic pain is associated with changes in oscillatory neural activity. Most commonly, an increase in theta activity is reported, and also an increase in alpha and beta activity (Sarnthein et al., 2006; Stern et al., 2006; Lim et al., 2016; Pinheiro et al., 2016; Ploner et al., 2017). Moreover, a recent recommendation in the development of safe and effective neurotherapeutics for pain emphasized the importance of the identification of objective biomarkers to help define pathophysiological subtypes of pain, evaluate target engagement of a therapy and predict therapeutic response (Davis et al., 2020). Electroencephalography (EEG)-based biomarkers of pain could be particularly useful to not only classify chronic pain, but also to individualize pain treatment and to serve as targets for neurotherapeutics. However, more work is required to identify accurate and clinically relevant EEG-based biomarkers (Ploner and May, 2018). Therefore, in this study we investigated the effects of low-frequency PEMS on cortical oscillatory activity in the theta, alpha and beta band. In a first experiment, two 10-min blocks of PEMS were compared to sham stimulation. In a second experiment, sensorimotor state-dependency of the effect of PEMS on cortical oscillatory activity was assessed, by comparing PEMS applied during MI (concurrent stimulation) to PEMS applied after MI (delayed stimulation). To assess the effect on cortical oscillatory activity, resting-state EEG was recorded before and after each stimulation condition. Finally, previous research has also demonstrated a significant positive relationship between the self-reported kinesthetic vividness of MI and the MI-induced change in corticomotor excitability (Williams et al., 2012; Vasilyev et al., 2017; Moriuchi et al., 2020) and intracortical excitability (Lebon et al., 2012). Therefore, the Kinesthetic and Visual Imaging Questionnaire (KVIQ) (Malouin et al., 2007) was included in experiment 2 to assess the participants’ MI ability, to explore if any relationship was present between visual and kinesthetic MI ability and the change in cortical oscillatory activity following state-dependent PEMS.

## Materials and Methods

### Participants

The study protocol was approved by the Ethical Committee of the Medical Faculty of the University of Tübingen. All participants provided written informed consent prior to participation. A screening questionnaire was completed to ensure that all participants met the inclusion criteria of the study. All participants were aged 18 or older, free of any neurological conditions and chronic pain conditions, had no history of drug misuse or sleep deprivation, and did not use any medication that could influence the assessments. Potential participants were excluded if they had participated in another neurostimulation study in the last 48 h. Right-handedness was confirmed with the Edinburgh Handedness Inventory (Oldfield, 1971). Participants were instructed to not consume any caffeinated drinks on the day of a study visit.

Experiment 1 consisted of two study visits. In a within-subject design, 26 participants completed the first session and twenty of these participants also completed the second session. Experiment 2 consisted of a single session that was completed by twenty participants. For the statistical analysis of experiment 1, the datasets of 2 of the 20 participants that completed both experimental sessions were excluded, resulting in a total of 18 datasets (mean age ± SD = 23.28 ± 5.00 years; 10 female); one dataset was excluded due to missing data and one dataset was excluded due to poor data quality. For experiment 2, the datasets of 2 participants were excluded from the statistical analysis, resulting in a total of 18 datasets (mean age ± SD = 23.78 ± 4.43 years; 10 female); one dataset was excluded due to technical issues and one dataset was excluded due to poor data quality.

### Procedure

#### Experiment 1

**FIGURE 1:**
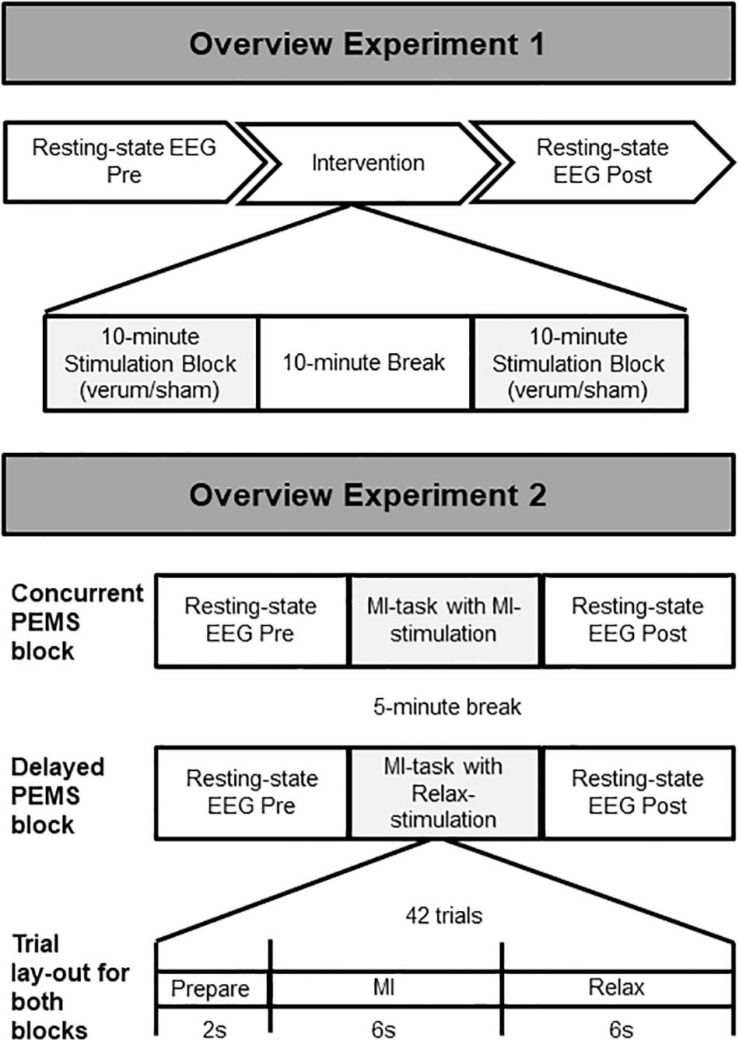
Overview of the study procedure. Experiment 1: Two stimulation conditions (verum and sham) were delivered during two separate stimulation sessions (session 1 = verum, session 2 = sham). The same overall procedure was followed for each session, starting with 5-min recording of resting-state EEG, followed by a total of 20 min of PEMS (verum) or sham stimulation, and finally another 5-min recording of resting-state EEG. Experiment 2: Two stimulation conditions (concurrent and delayed PEMS) were delivered during a single session, in two separate blocks. Order of stimulation conditions was randomized. In the concurrent PEMS condition, PEMS was applied during the MI phase of the trial. In the delayed PEMS condition, PEMS was applied during the Relax phase of the trial.

In a within-subject design, participants attended the lab for two study visits to undergo two stimulation conditions in a non-randomized order: verum stimulation (visit 1) and sham stimulation (visit 2), with a minimum of 2 weeks in between study visits. For each visit, first 5 min of resting-state EEG was recorded. This was followed by two 10-min blocks of PEMS/sham stimulation with a 10-min break between blocks, in line with the treatment recommendations of the device manufacturer (Bomedus GmbH). Another 5 min of resting-state EEG was recorded after the two stimulation blocks (Figure 1). Each 5-min resting-state recording consisted of ten 30-s intervals of eyes open (EO) and eyes closed (EC). Participants received auditory cues to instruct them to open/close their eyes and were asked to focus on the fixation cross on the computer screen for the EO condition to minimize eye movements.

#### Experiment 2

Experiment 2 consisted of a single study visit. Two stimulation conditions were applied in separate blocks in a randomized order (Figure 1). Each block consisted of 42 trials of MI; participants were asked to imagine extension of the fingers of the left hand. Participants received visual and auditory cues as guidance for the MI task. For each MI trial, a 2-s preparation phase (“Ready”), was followed by a 6-s MI phase (“Imagine”), and finally a 6-s rest phase (“Relax”). For one stimulation condition PEMS was applied during the MI phase of each trial (concurrent PEMS), for the other stimulation condition PEMS was applied after MI during the rest phase of each trial (delayed PEMS). Each stimulation block had a duration of ∼10 min and there was a 5-min break between blocks. Five minutes of resting-state EEG was recorded before and after each stimulation block. Participants were asked to keep their eyes opened and focused on the fixation cross on the computer screen for the entire 5-min recording. In addition, all participants completed the Kinesthetic and Visual Imaging Questionnaire (KVIQ) (Malouin et al., 2007) to assess their ability to feel and visualize imagined movements.

### PES With the Matrix Electrode (PEMS)

**FIGURE 2:**
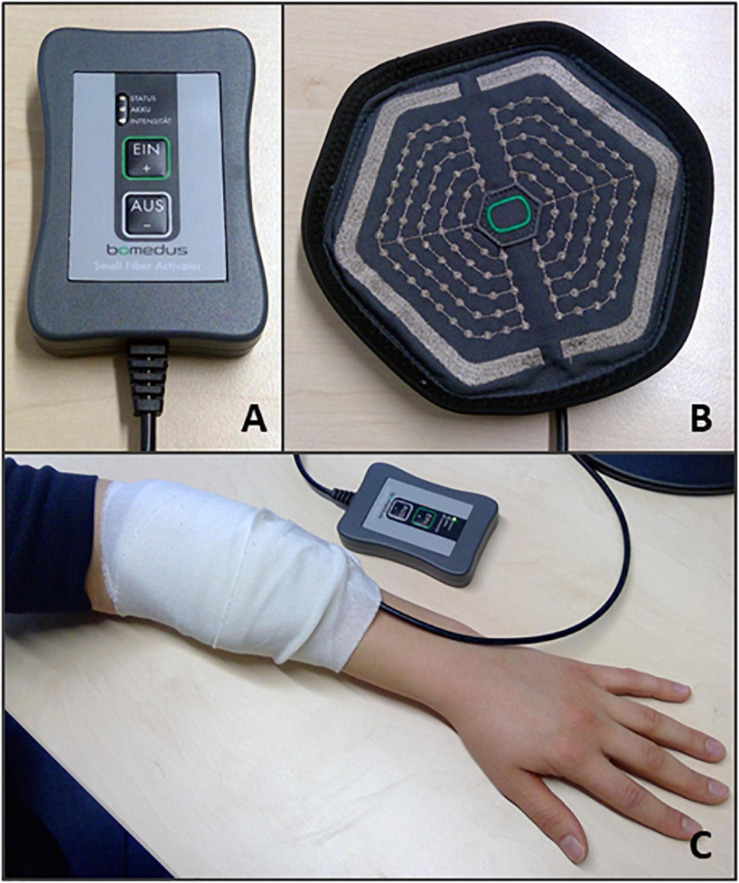
PEMS set up. The following equipment was used to deliver the PEMS for both experiments: **(A)** The PEMS stimulator (The Small Fiber Activator; Bomedus GmbH); **(B)** A circular matrix electrode (Bomedus GmbH). **(C)** Shows the placement of the matrix electrode on the left forearm. A bandage was wrapped around the electrode to ensure optimal contact between the electrode and the skin.

For both experiments PES was applied using a matrix array electrode (Bomedus GmbH, Germany) that was placed over the left Extensor Digitorum Communis (EDC) muscle. A circular matrix electrode was used with a diameter of 15 cm (Figure 2). The Bomedus stimulator used, (The Small Fiber Activator; Bomedus GmbH) generated monopolar rectangular pulses with a width of 200 μs and a frequency of 4 Hz. A bandage was wrapped around the matrix electrode to ensure optimal contact between the electrode and the skin.

#### PEMS Experiment 1

For the verum stimulation condition PEMS was applied continuously (for two blocks of 10 min) at an intensity just below pain threshold, which resulted in a sensation of intense prickling under the electrode. To identify the pain threshold intensity participants were asked to gradually increase the stimulation intensity themselves and to select an intensity as high as possible before becoming painful. Participants were allowed to increase/decrease the intensity during the stimulation as well, to ensure that a sensation of intense prickling (but no pain) remained present throughout.

No stimulation was applied during the sham condition. Here participants were asked to gradually increase the stimulation intensity themselves until they first detected a prickling sensation. Then an intensity was chosen that was just below the sensory threshold. Subjects were informed that during this session the stimulation would be applied at an intensity just below the sensory threshold and would thus not be noticeable. As soon as the experiment started, the stimulator was automatically turned off, without the participant knowing.

#### PEMS Experiment 2

For experiment 2, PEMS was applied intermittently either during the 6-s MI phase of each trial (the concurrent PEMS condition) or the 6-s rest phase of each trial (the delayed PEMS condition). Stimulation intensity was set individually, following the same procedure as for the verum stimulation (experiment 1) to ensure participants experienced a strong prickling (but not painful) sensation during the stimulation. The stimulation intensity was set separately before each of the two blocks of the experiment.

### EEG Recordings

Electroencephalography was recorded using a 64-channel actiCAP combined with BrainAmp DC amplifiers (Brain Products GmbH, Germany). Impedances were kept below 25 kΩ. The AFz electrode location was used as the ground electrode and the FCz electrode location as the reference electrode.

### Kinesthetic and Visual Imaging Questionnaire

For experiment 2, all participants completed the Kinesthetic and Visual Imaging Questionnaire (KVIQ) (Malouin et al., 2007). The questionnaire includes two subscales, the Visual Imagery Scale (VIS) and the Kinesthetic Imagery Scale (KIS) that contain 10 items each. For each item participants are asked to first perform a movement (e.g., elbow flexion) and then imagine performing the same movement. Next, participants rate on a 5-point scale: (i) the clarity of the visual image (1 = no image, 5 = image as clear as seeing); or (ii) the intensity of the sensations associated with the imagined movement (1 = no sensation, 5 = as intense as executing the action). In line with Malouin et al. (2008), items involving limb movement were tested on both sides. This resulted in a total of 17 ratings for each scale and a maximum sum score of 85 for each scale.

### EEG Analysis

All EEG recordings were analyzed in MATLAB (The Mathworks, Inc., Natick, MA, United States) using custom built code and the Fieldtrip toolbox (Oostenveld et al., 2011). For both experiments the same pre-processing and frequency analysis procedure was applied. For each recording, a 1 Hz high-pass filter (4th order Butterworth) was applied on the continuous data and the signal was re-referenced to the average reference. Next, the continuous EEG data was segmented into consecutive 1-s epochs. Any bad channels were interpolated. Finally, epochs containing artifacts were rejected using an automated artifact detection procedure. Any epochs containing data with a range >200 μV were rejected. The re-referencing to an average-reference was repeated after the artifact rejection procedure to ensure that for the final re-referencing the interpolated channels were included instead of the original noisy channels.

Whereas for experiment 1 resting-state EEG with EO and EC was collected, it was decided to only use the EO data for further analysis, since the closing of the eyes affects the power of ongoing alpha activity and pooling across EO and EC resting-state data would introduce variance of alpha activity. This also ensured that for both experiments the same type of resting-state data was analyzed (i.e., EO data only), as experiment 2 only included EO resting-state data. Frequency analysis was performed using the Fieldtrip toolbox (Oostenveld et al., 2011). Average EEG power was calculated using the fast Fourier transform (FFT) with a single Hanning taper for three frequency bands of interest: the theta (4–7 Hz), alpha (8–13 Hz), and beta band (15–30 Hz). For both experiments, average EEG power for the theta, alpha, and beta band was calculated for the resting-state EEG recorded before and after each stimulation condition. Finally, a change from baseline score was calculated for each stimulation condition and each frequency band of interest ((EEG power post – EEG power pre)/EEG power pre) and used for the statistical analysis. For experiment 1, this resulted in a comparison of the change from baseline score for verum stimulation versus sham stimulation for theta, alpha, and beta power separately. For experiment 2, this resulted in a comparison of the change from baseline score for concurrent PEMS (PEMS during the MI phase) versus delayed PEMS (PEMS after MI, during the rest phase).

### Statistical Analysis

To assess a statistical difference between verum and sham stimulation (experiment 1) and concurrent and delayed stimulation (experiment 2), non-parametric tests were used. Non-parametric tests were chosen over parametric tests as there was evidence that the assumption of normality was not met, based on the Kolmogorov-Smirnov tests of normality and the kurtosis and skewness z-scores. Additionally, non-parametric permutation tests were used as they allow for the estimation of the statistical significance of spatial clusters. For experiment 1, the Kolmogorov-Smirnov tests of normality and the kurtosis and skewness z-scores were calculated for the difference scores in change from baseline for theta, alpha, and beta power comparing verum and sham stimulation. The same was applied for experiment 2, here for the difference scores comparing concurrent and delayed stimulation. For experiment 1 and 2 together a total of 18 tests were calculated to assess normality. More than 50% (61%) of these tests had a *p* < 0.05. For the PEMS stimulation levels applied, no strong evidence for a violation of the assumption of normality was present. For experiment 1, the kurtosis and skewness *z*-scores and the Kolmogorov-Smirnov test of normality were calculated for the difference score of the PEMS level of block 1 and block 2. For experiment 2, the kurtosis and skewness *z*-scores and the Kolmogorov-Smirnov test of normality were calculated for the difference score of the PEMS level of concurrent and delayed stimulation. Here, out of the total of 6 tests performed, less than 50% (33%) of tests had a *p* < 0.05. Therefore, for the descriptive statistics parametric tests were used.

#### Global EEG Power Changes

For both experiments, first, global EEG power changes were assessed (i.e., EEG power averaged over all EEG electrodes) using SPSS version 26 (IBM Corp, Armonk, NY, United States). Wilcoxon signed-rank tests were used to test for a significant difference in the change from baseline scores of global EEG power for (1) verum versus sham stimulation, and (2) concurrent PEMS versus delayed PEMS. This was done for global theta, alpha, and beta power separately, resulting in the calculation of three Wilcoxon signed-rank tests for each experiment. The exact 2-sided significance was reported.

#### Cluster-Based Permutation Tests

In a second step, cluster-based permutation tests (Maris and Oostenveld, 2007) were carried out using the Fieldtrip toolbox (Oostenveld et al., 2011) in MATLAB, to identify clusters where a significant difference in EEG power was present for each stimulation condition. Cluster-based permutation tests offers a more data-driven approach to identify patterns in oscillatory neural activity without strong prior assumptions and are well suited to control for the problem of multiple comparisons (Maris and Oostenveld, 2007). As there was no previous research available on the effects of PEMS on oscillatory neural activity to inform a specific frequency range and/or scalp region of interest in the present study, here we included the full range of EEG frequencies (4–30 Hz, i.e., the theta, alpha, and beta band together) and each individual EEG electrode for each cluster-based permutation test, instead of running a test for each frequency band separately.

The general cluster-based permutation test procedure that was applied for all tests in this study was as follows: (1) for every frequency-electrode pair (64 electrodes, frequency range 4–30 Hz) a repeated measures *t*-statistic was calculated (e.g., to compare EEG power before and after verum stimulation), to be used later to calculate the cluster-level test statistic; (2) samples were selected using the uncorrected threshold of *p* < 0.05 and the selected samples were clustered in connected sets based on spatial and spectral adjacency; (3) cluster-level statistics were calculated by taking the sum of the *t*-values within each identified cluster; (4) to calculate the significance probability for the cluster-level test statistic the Monte Carlo method was used. A permutation was performed 2000 times (i.e., 2000 random partitions) to generate a random-partition-based cluster-level test statistic and (5) corrected *p*-values were calculated by comparing the values of the cluster-level statistics of the observed data against the distribution of the cluster-level test statistic based on the 2000 permutations.

For experiment 1, two cluster-based permutation tests were carried out to compare EEG power for the pre- and post-stimulation resting-state recordings. For the verum and sham condition separately, a comparison of resting-state EEG power pre- and post-stimulation was carried out to assess any significant changes following stimulation. The same approach was used for experiment 2, for the concurrent PEMS and delayed PEMS condition, i.e., two tests were carried out to assess any pre-post EEG power differences for each stimulation condition separately. Because we performed two statistical tests for each experiment, we used Bonferroni-correction for multiple comparison, rendering an effective alpha-threshold of 0.025. Finally, for each cluster-based permutation test, a Cohen’s *d* effect size for dependent samples was calculated for each cluster with an uncorrected *p*-value < 0.05 (i.e., each cluster that was significant before correction for multiple comparison), by dividing the mean difference by the standard deviation of the difference (Lakens, 2013). To do this, the clustered channels which exhibited a difference were selected and the mean EEG power and standard deviation were calculated for these channels across the clustered frequencies which exhibited a difference.

#### Correlations

For experiment 2, a number of correlations were calculated to assess whether a change in EEG power in response to PEMS was related to the participant’s MI capability. The sum score for the KIS and VIS were each correlated with the change from baseline scores for global theta, alpha, and beta power, for the concurrent PEMS and the delayed PEMS condition separately. I.e., 6 correlations were calculated for the VIS subscale and 6 correlations for the KIS subscale. The Bonferroni corrected significance level of 0.0083 was used.

## Results

### Descriptive Statistics

The PEMS stimulator used in both experiments (The Small Fiber Activator; Bomedus GmbH), has a maximum output current of 40 mA and comes with 30 pre-set stimulation levels of which the participants could select their individual stimulation level.

For experiment 1, taking into account any adjustments made by the participants during the stimulation, the average stimulation level [mean (SD)] for verum stimulation was 14.94 (5.70) for stimulation block 1 and 15.86 (5.62) for stimulation block 2. A repeated measures *t*-test showed that there was no significant difference in stimulation level for block 1 compared to block 2 (*t* = −1.94, *p* = 0.069, *N* = 18).

For experiment 2, the average stimulation level used was 21.22 (7.62) for the concurrent PEMS condition and 20.39 (7.84) for the delayed PEMS condition. A repeated measures *t*-test showed that there was no significant difference in stimulation level between the two conditions (*t* = 1.16, *p* = 0.26, *N* = 18).

### Global EEG Power

**FIGURE 3:**
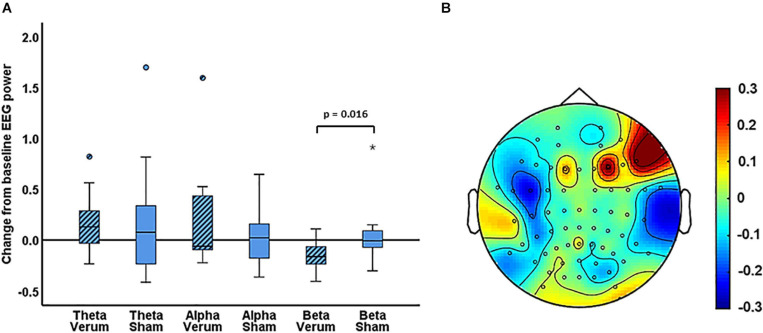
**(A)** Boxplots of the change from baseline scores ((EEG power post – EEG power pre)/EEG power pre) for the verum and sham PEMS condition, and for the theta, alpha, and beta band, separately. The ° and the * in the figure indicate outliers. **(B)** A topography plot of the average change from baseline of beta power for the PEMS condition (verum stimulation).

For experiment 1, the Wilcoxon signed rank tests comparing the change from baseline scores of global EEG power ((EEG power post – EEG power pre)/EEG power pre) for sham versus verum stimulation, showed a significant difference in the change from baseline for beta power (*Z* = −2.37, *p* = 0.016, *N* = 18). For beta power, the median change from baseline was −16% for verum stimulation and −0.54% for sham stimulation (Figure 3). No significant difference between verum and sham was found for the theta band (*Z* = −0.94, *p* = 0.37) or the alpha band (*Z* = −0.59, *p* = 0.56). The median change from baseline was 13% and 7.80% for the theta band and −18% and −5.88% for the alpha band, for verum and sham stimulation, respectively.

For experiment 2, the Wilcoxon signed rank tests comparing the change from baseline of global EEG power for concurrent PEMS versus delayed PEMS, did not identify a significant change of global power in any of the frequency bands (theta: *Z* = −0.46, *p* = 0.67; alpha: *Z* = −0.54, *p* = 0.61; and beta: *Z* = −0.065, *p* = 0.97). The median change from baseline was −0.57% and 2.83% for the theta band, 0.97% and −5.89% for the alpha band, and 0.41% and 4.63% for the beta band, for concurrent and delayed PEMS, respectively.

### Cluster-Based Permutation Tests

#### Experiment 1: Verum and Sham Stimulation

**FIGURE 4:**
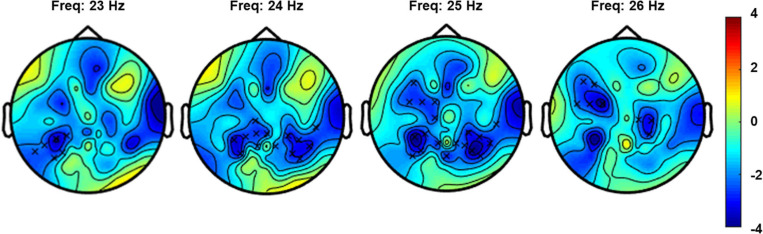
Topoplot for the cluster-based permutation test that compared resting-state EEG power (4–30 Hz) before and after verum stimulation. The plot shows the negative cluster (indicated by the “x” markers) that demonstrated a significant difference between EEG power before and after PEMS, plotted on top of the *t*-statistic of the difference as calculated for every frequency-electrode pair (repeated-measures *t*-test). The cluster showed a significant difference most pronounced over bilateral central and left frontal regions and in the frequency range of 23–26 Hz.

The non-parametric cluster-based permutation test for the verum stimulation condition, assessing a difference in resting-state EEG power before and after verum stimulation, indicated a significant difference (*p* = 0.018). This corresponded to a negative cluster in the beta frequency band that was most pronounced over bilateral central and left frontal sensors (Figure 4). The Cohen’s *d* effect size for this cluster was 0.41 (for EEG power averaged across the frequency range of 23–26 Hz and including the following 27 clustered electrodes: FT7, F5, F7, FC1, FC3, FC5, C1, C2, C4, CPz, CP1, CP3, CP4, CP5, CP6, T8, TP7, TP8, TP9, P1, P2, P4, P5, P6, P7, P8, and POz). The non-parametric cluster-based permutation test for sham stimulation did not show any significant difference for pre- and post-sham EEG power.

#### Experiment 2: Concurrent and Delayed PEMS

**FIGURE 5:**
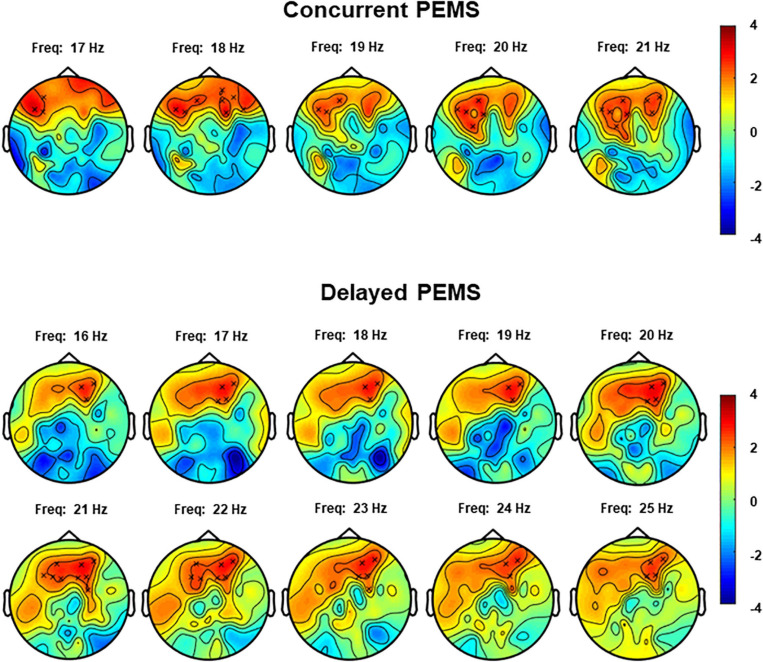
Topoplots for the cluster-based permutation test that compared resting-state EEG power (4–30 Hz) before and after concurrent and delayed stimulation. Each plot shows the largest positive cluster (indicated by the “x” markers) that demonstrated a difference between EEG power before and after PEMS, plotted on top of the t-statistic of the difference as calculated for every frequency-electrode pair (repeated-measures *t*-test). The cluster showed a difference most pronounced over frontal regions and in the frequency range of 17–21 Hz for concurrent PEMS and 16–25 Hz for delayed PEMS.

The non-parametric cluster-based permutation test for the concurrent PEMS condition, suggested a trend of a difference in resting-state EEG power comparing before and after concurrent stimulation (*p* = 0.029). This corresponded to a positive cluster in the beta frequency range that was most pronounced over frontal sensors (Figure 5). However, this did not survive correction for multiple comparisons, i.e., this was not significant using the corrected significance level of 0.025. The Cohen’s *d* effect size for this cluster was 0.28 (for EEG power averaged across the frequency range of 17–21 Hz and including the following 11 clustered electrodes: AF3, AF4, AF7, AF8, Fp2, F1, F4, F5, F7, F8, and FC3).

The non-parametric cluster-based permutation test for delayed PEMS similarly suggested a trend of a difference in resting-state EEG power comparing before and after delayed stimulation (*p* = 0.039). This corresponded to a positive cluster in the beta frequency range that was most pronounced over frontal sensors (Figure 5). However, again, this did not survive correction for multiple comparisons, i.e., this was not significant using the corrected significance level of 0.025. The Cohen’s *d* effect size for this cluster was 0.39 (for EEG power averaged across the frequency range of 16–25 Hz and including the following 9 clustered electrodes: AF3, AF4, AF8, F1, F2, F3, F4, F5, and FC4).

### Correlations

For the two subscales of the KVIQ, the average sum score was 62.94 (12.31) with a range of 36–76 for the VIS and the average sum score for the KIS was 60.61 (15.31) with a range of 20–76. For the concurrent PEMS condition, a correlation between the VIS sum score and the change from baseline score of beta power was identified (*R* = −0.60, *p* = 0.009). However, this did not survive correction for multiple comparisons (the corrected significance level was 0.0083). No significant correlations were found for the delayed PEMS condition. For the KIS subscale, no significant correlations were found either (Table 1).

**TABLE 1:** Overview of the results of the correlation analysis.

	**VIS sum score (*N* = 18)**	**KIS sum score (*N* = 18)**
Concurrent PEMS – theta	*r* = 0.092, *p* = 0.72	*r* = 0.55, *p* = 0.017
Concurrent PEMS – alpha	*r* = 0.005, *p* = 0.98	*r* = 0.22, *p* = 0.38
Concurrent PEMS – beta	*r* = −0.60, *p* = 0.009	*r* = −0.26, *p* = 0.30
Delayed PEMS – theta	*r* = 0.074, *p* = 0.77	*r* = 0.19, *p* = 0.44
Delayed PEMS – alpha	*r* = 0.15, *p* = 0.55	*r* = 0.087, *p* = 0.73
Delayed PEMS – beta	*r* = −0.47, *p* = 0.052	*r* = −0.43, *p* = 0.072

*Correlations were calculated for the sum score of the VIS and the KIS, and the change from baseline scores of EEG power in the theta, alpha, and beta range, both for the concurrent and delayed PEMS condition. A correlation between the VIS sum score and the change from baseline score of beta power was identified for the concurrent PEMS condition. However, this did not survive correction for multiple comparisons (the corrected significance level was 0.0083).*

## Discussion

Cortical oscillatory activity has been proposed as a promising target for the development of novel pain neurotherapeutics (Jensen et al., 2008; Ploner and May, 2018). A novel neurostimulation approach delivering low-frequency PES via a matrix electrode, has been shown particularly effective to reduce deep pain sensitivity (Mücke et al., 2014) and has been applied to reduce pain in patients with ongoing cancer pain (Mücke et al., 2018). The present study investigated for the first time the effects of PEMS on cortical oscillatory activity. Experiment 1 showed that low-frequency PEMS (4 Hz) resulted in a significantly larger reduction of global beta power compared to sham stimulation; the median change from baseline was −16% for PEMS and −0.54% for sham. No significant difference was identified for the theta and alpha frequency band. The cluster-based permutation test comparing resting-state EEG power before and after PEMS also resulted in a significant difference, which corresponded to a negative cluster in the beta frequency band most pronounced over bilateral central and left frontal sensors. This study provides initial evidence that low-frequency PEMS results in a widespread reduction of resting-state beta power following 20 min of stimulation, most prominent over central and frontal scalp regions.

Previously, a small number of studies also showed changes in oscillatory neural activity for PES, with varying outcomes. One study that investigated the effects of high-frequency-low-intensity TENS and low-frequency-high intensity TENS in 80 healthy pain-free participants showed that only the low-frequency-high-intensity TENS resulted in a long-lasting enhancement of ongoing alpha activity in the primary sensorimotor cortex compared to sham (Peng et al., 2019). Another study showed that following 20 min of conventional TENS at 70 Hz an increase of posterior theta power was present in patients with fibromyalgia (Yüksel et al., 2019). In addition, an increase in anterior alpha power and a decrease in posterior alpha power was found. However, it should be noted that this study did not include a sham condition. Insausti-Delgado et al. (2021) showed that neuromuscular electrical stimulation (NMES) over the wrist extensors resulted in an event-related desynchronization of sensorimotor alpha and beta oscillations, which was influenced by stimulation intensity. During high-intensity NMES (above motor threshold) a significantly larger alpha and beta desynchronization was present than during low- and medium-intensity stimulation (below motor threshold). Finally, Tu-Chan et al. (2017) investigated the potential of somatosensory electrical stimulation to improve hand function in individuals with acquired brain injury. In this pilot study, TENS was applied simultaneously over the median, ulnar, and radial nerve at an intensity that induced a clear and strong sensation but no pain or visible muscle contractions. A single 2-h session resulted in a significant improvement of hand function and a significant reduction of resting-state delta and theta power. Moreover, the improvement of finger movement was significantly correlated with the combined change of theta and alpha power over ipsilesional sensorimotor regions.

In the present study, we applied low-frequency PES with a matrix electrode to preferentially stimulate the nociceptive afferents in the superficial skin layer and found an increase in resting-state beta power over central and frontal scalp regions, following 20 min of PEMS. Thus, this study provides initial evidence for a role of beta oscillations in the cortical effects of PEMS. Moreover, the combined findings of the present study and the previous studies identifying a change in oscillatory neural activity in response to TENS and NMES, suggest that the choice of PES electrode (e.g., targeting tactile/nociceptive afferents) may have an influence on the effect of PES on cortical oscillatory activity, along with the applied stimulation parameters such as stimulation frequency and intensity. However, further studies are necessary to directly compare different electrode types, stimulation frequencies and intensities (Nilsson et al., 2003; Jung et al., 2009).

Secondly, the present study also offered an initial exploration of state dependency of the effects of PEMS on cortical oscillatory activity. Using MI to modulate sensorimotor brain activity, we assessed state-dependency of PES by comparing two stimulation conditions: (1) concurrent PEMS, i.e., PEMS applied during a state of MI-induced oscillatory desynchronization (sensorimotor activation); and (2) delayed PEMS, i.e., PEMS applied directly after MI during a state of oscillatory synchronization/rebound (sensorimotor deactivation) (Pfurtscheller and Neuper, 1997; Fadiga et al., 1999; Pfurtscheller et al., 2005, 2006). This study did not identify a significant difference between concurrent and delayed PEMS for the change from baseline of global theta/alpha/beta power, i.e., the timing of intermittent bursts of low-frequency PEMS with respect to the underlying sensorimotor brain state, did not influence the change from baseline of global cortical oscillatory activity. In addition, no significant correlations between kinesthetic and visual MI ability (KVIQ) and changes in oscillatory neural activity following state-dependent PEMS were identified. In previous studies, sensorimotor state-dependency of the effects of TMS and peripheral stimulation on corticospinal excitability (Saito et al., 2013; Kaneko et al., 2014; Kraus et al., 2016a) and intracortical motor circuits has been demonstrated (Guggenberger et al., 2018; Kraus et al., 2018; Ziegler et al., 2019).

Whereas sensorimotor state-dependency of PES has been shown for studies targeting the motor system, the present study did not show a similar state-dependency of the effects of PES on resting-state cortical oscillatory activity, when using a matrix electrode that preferentially stimulates nociceptive afferents in the skin. This is somewhat in contrast with the findings of Corbet et al. (2018), who showed that the online effects of MI on sensorimotor desynchronization were enhanced when MI was combined with NMES at sensory threshold intensity. When sensory threshold NMES was applied without MI, no significant sensorimotor desynchronization was present. However, these findings were based on changes in sensorimotor activation during the MI task, whereas the present study assessed changes comparing resting-state activity before and after stimulation. Thus, further investigation of the online effects of sensorimotor state-dependent PES could be a useful direction for future studies.

The combined findings of experiment 1 and 2, however, do suggest that adding the cognitive task of MI to PEMS may have a more general influence on the effects of PEMS on oscillatory activity. Whereas the cluster-based permutation tests for experiment 1 showed a decrease of central and frontal resting-state beta power following 20 min of continuous PEMS, this was not the case in experiment 2. Here, PEMS was applied intermittently and together with a MI task. Moreover, the cluster-based permutation tests from experiment 2 provided preliminary evidence to suggest an increase of beta power over frontal scalp regions, both after concurrent and delayed PEMS (*p* < 0.05), albeit this finding did not survive correction for multiple comparisons. Thus, the combined findings of experiment 1 and 2 suggest a potential influence of the attentional or cognitive state, induced by the MI task, on low-frequency PEMS.

Further support for an influence of attentional/cognitive factors on PEMS effects comes from the finding that PEMS compared to sham modulated beta power not only in central but also frontal regions and the initial indication that PEMS combined with MI modulates beta power in frontal regions in particular. Although traditionally, beta oscillations have been associated with sensorimotor function and motor control, more recently, beta oscillations have been investigated in a wider range of cortical areas and have been implicated in a wider range of cognitive functions (Spitzer and Haegens, 2017). In particular, beta oscillations have been associated with top-down processing functions (Engel and Fries, 2010). For example, coherence in the beta frequency range between the frontal and parietal cortex was found in particular for top-down control of attention (compared to bottom-up control of attention) in monkeys (Buschman and Miller, 2007). In another study, humans showed a dissociation in beta oscillatory changes using a motor go/no-go paradigm (Alegre et al., 2004). A central decrease followed by an increase of beta activity was associated with movement preparation and execution, whereas a frontal increase of beta was associated with decision making and motor inhibition. Similarly, Wagner et al. (2016) showed that two distinct beta oscillatory networks were involved in motor adjustments during gait adaptation: suppression of beta power in central and parietal regions (motor execution) and an increase of beta power in prefrontal regions (cognitive top-down control).

Finally, some previous studies have also demonstrated an influence of attention and cognitive state on the effects of neurostimulation. For example, Sarkar et al. (2014) found that transcranial direct current stimulation (tDCS) over the prefrontal cortex improved reaction times for a simple arithmetic task in individuals with high anxiety related to mathematics, whereas individuals with a low anxiety level had impaired reaction times. Another study showed that the effect of tDCS applied over the parietal cortex on visual working memory was influenced both by task difficulty and participants’ working memory capacity (Jones and Berryhill, 2012). For pain, it has been shown that the effect of alternating current stimulation (tACS) at alpha frequency on pain perception is influenced by expectations about pain (Arendsen et al., 2018); a reduction of pain was found when participants were uncertain about the intensity of an upcoming painful stimulus, but not when the intensity of an upcoming stimulus was predictable. However, it should be emphasized that no definitive conclusions can be drawn based on the comparison of the results of experiment 1 and 2, since the two experiments had differences in some stimulation parameters (experiment 1: a total of ∼20 min of continuous stimulation; experiment 2: ∼10 min of intermittent stimulation) that may have influenced the findings. Thus, well-controlled studies are recommended to further investigate the potential influence of a cognitive task on the effects of low-frequency PEMS.

The present study in pain-free participants showed a modulation of resting-state oscillatory activity in the beta band specifically. Chronic pain is associated with changes in cortical oscillatory activity in a variety of frequency bands, including the beta band. In particular, an increase of beta power in frontal regions has been demonstrated (Sarnthein et al., 2006; Stern et al., 2006; Lim et al., 2016; Ploner et al., 2017). PES using the matrix electrode to modulate frontal beta power may therefore offer a novel direction in the application of PES to reduce chronic pain. The initial suggestion of this study, that the effects of low-frequency PEMS may be influenced by the presence of a cognitive task (albeit in pain-free participants), is also of interest to the application of PES interventions to reduce chronic pain. To improve the efficacy of neurostimulation interventions to manage chronic pain, it is important to take into account inter- and intra-individual factors such as cognitive, psychological, and neurophysiological state (Li et al., 2015; Fertonani and Miniussi, 2017). Thus, further investigations on the influence of adding a cognitive task such as MI to PES interventions to reduce chronic pain should be considered. In addition, adding a cognitive task such as MI introduces an element of active participation to any intervention. Active patient engagement in the therapeutic context is key in achieving lasting clinical improvements (Lequerica and Kortte, 2010; Blank et al., 2014). Ultimately, improving our understanding of the influence of these attentional/cognitive factors on the effects of PES may improve the efficacy of PES interventions to reduce chronic pain. However, future investigations should also include measures of pain experience, to assess whether the modulation of beta activity in associated with a reduction in pain. Whereas the potential of PEMS to reduce pain in healthy pain-free participants has been demonstrated previously (Mücke et al., 2014), no simultaneous assessment of changes in oscillatory neural activity and pain experience following PEMS has been carried out yet. Confirming a relationship between the modulation of beta power and pain experience would be an important next step in the confirmation of beta power as a potential biomarker for low-frequency PEMS, and in line with the recent recommendations of the importance of identifying objective biomarkers for the development of safe and effective neurotherapeutics for pain (Davis et al., 2020).

Another factor deserving further investigation is whether any effects following (state-dependent) PES remain present for a longer period after stimulation. The present study focused on changes in oscillatory neural activity in the period immediately after PEMS and did not include any longer-term assessments of change in oscillatory neural activity (e.g., 30 min after stimulation). Some previous studies investigating cortical effects of low-frequency PES have shown a longer-term reduction of SEP amplitude (up to 30–60 min post-stimulation) (Jung et al., 2009, 2012). However, less is known about any longer-lasting effects of PES on oscillatory neural activity, especially for PES combined with MI. Previous studies demonstrated an online effect of PES combined with MI on corticospinal excitability (Saito et al., 2013; Kaneko et al., 2014) and sensorimotor desynchronization (Corbet et al., 2018). When a combined TMS and peripheral stimulation protocol was applied sensorimotor state-dependently, a modulation of corticospinal excitability and cortical motor maps survived a depotentiation task with voluntary muscle contraction after the stimulation indicating robustness (Guggenberger et al., 2018; Kraus et al., 2018). When considering the implementation of state-dependent low-frequency PES as a pain intervention, it is critical that we gain a better understanding of any longer-lasting effects of PES and the specific stimulation parameters that are most effective to inducing a plastic change in central nociception and the perception of pain.

Finally, a recent study showed that MI together with NMES at sensory threshold resulted in a larger desynchronization of sensorimotor oscillatory activity and a significant enhancement of brain connectivity patterns (compared to MI accompanied by visual feedback) (Corbet et al., 2018). A significantly higher connectivity was found for MI with NMES in the fronto-parietal network, including the associative somatosensory cortex, premotor cortex and supplementary motor area, and the primary motor cortex. These observations confirmed previous findings applying MI with visual or proprioceptive feedback (Vukeliæ and Gharabaghi, 2015): Both feedback modalities activated a distributed functional connectivity network of coherent oscillations. However, proprioceptive feedback was more suitable than visual feedback to entrain the motor network architecture (e.g., beta-band and theta-band activity in bilateral fronto-central regions and left parieto-occipital regions, respectively) during the interplay between motor imagery and feedback processing, thus resulting in better volitional control of regional brain activity. Therefore, the inclusion of connectivity analysis would be recommended for future studies investigating the cortical effects of brain state-dependent PES, to gain further insight on the effects of PES on the interaction between somatosensory and motor brain regions.

## Conclusion

This study investigated the effects of low-frequency PEMS on cortical oscillatory activity in the theta, alpha and beta band. Secondly, sensorimotor state-dependency of the effect of PEMS on cortical oscillatory activity was assessed using a MI task. Low-frequency PEMS (4 Hz) resulted in a significantly larger reduction of global beta power compared to sham stimulation after the stimulation period. This reduction was most pronounced over central and frontal scalp regions. Furthermore, there was some initial evidence to suggest an influence of MI on the effect of PEMS. Following PEMS combined with a MI task no decrease of global beta power was present. Instead, the results provide preliminary evidence for an increase of frontal beta power following both concurrent and delayed PEMS, although there was no significant difference between these two conditions. This study provides novel evidence for supraspinal effects of low-frequency PEMS and an initial indication that the presence of a cognitive task such as MI may influence the effects of PEMS on beta activity.

## Data Availability Statement

The raw data supporting the conclusions of this article will be made available by the authors, without undue reservation.

## Ethics Statement

The studies involving human participants were reviewed and approved by the Ethical Committee of the Medical Faculty of the University of Tübingen. The patients/participants provided their written informed consent to participate in this study.

## Author Contributions

AG, TW, and RG: conceptualization and study design. MZ: recruitment and data collection. LA, RG, and MZ: analysis. LA: writing – original draft preparation. All authors writing – review and editing.

## Conflict of Interest

TW is the founder and CEO of Bomedus GmbH, the company that developed the stimulation device used in this study. The remaining authors declare that the research was conducted in the absence of any commercial or financial relationships that could be construed as a potential conflict of interest. The reviewer EL-L declared a past co-authorship with one of the authors AG to the handling editor.
